# Competitive Mirror Image Phage Display Derived Peptide Modulates Amyloid Beta Aggregation and Toxicity

**DOI:** 10.1371/journal.pone.0147470

**Published:** 2016-02-03

**Authors:** Stephan Rudolph, Antonia Nicole Klein, Markus Tusche, Christine Schlosser, Anne Elfgen, Oleksandr Brener, Charlotte Teunissen, Lothar Gremer, Susanne Aileen Funke, Janine Kutzsche, Dieter Willbold

**Affiliations:** 1 Institute of Complex Systems, Structural Biochemistry (ICS-6), Research Centre Jülich, 52425 Jülich, Germany; 2 Fakultät Angewandte Naturwissenschaften, Hochschule für angewandte Wissenschaften Coburg, 96450 Coburg, Germany; 3 Institut für Physikalische Biologie, Heinrich-Heine-Universität Düsseldorf, 40225 Düsseldorf, Germany; 4 Neurochemistry Laboratory and Biobank, VU University Medical Center Amsterdam, The Netherlands; Universitat Autònoma de Barcelona, SPAIN

## Abstract

Alzheimer´s disease is the most prominent type of dementia and currently no causative treatment is available. According to recent studies, oligomeric species of the amyloid beta (Aβ) peptide appear to be the most toxic Aβ assemblies. Aβ monomers, however, may be not toxic per se and may even have a neuroprotective role. Here we describe a competitive mirror image phage display procedure that allowed us to identify preferentially Aβ_1–42_ monomer binding and thereby stabilizing peptides, which destabilize and thereby eliminate toxic oligomer species. One of the peptides, called Mosd1 (monomer specific d-peptide 1), was characterized in more detail. Mosd1 abolished oligomers from a mixture of Aβ_1–42_ species, reduced Aβ_1–42_ toxicity in cell culture, and restored the physiological phenotype in neuronal cells stably transfected with the gene coding for human amyloid precursor protein.

## Introduction

Dementia counts for more than 30 million patients worldwide. Alzheimer´s disease (AD) is the most frequent type of dementia and therefore contributes largely to this number [1, 2]. Because age is the most important risk factor for AD, the number of AD patients is expected to increase further.

AD is a progressive type of dementia with symptoms like memory loss, apathy, depression, anxiety and behavioral changes as well as neuropathological hallmarks including inflammation, neuronal cell death and loss of brain mass [2–6]. To date, AD can only be treated palliatively and symptomatically, although with only limited success. Throughout our life time, Aβ is constantly produced from the amyloid precursor protein (APP) by β- and γ-secretases [7, 8].

Currently, soluble Aβ oligomers are assumed to be the major toxic species in AD [9–11]. Aβ oligomers were shown to account already at nanomolar concentrations for neuronal atrophy, induction of synaptic dysfunction, formation of pores and thereby disturbance of cellular ion homeostasis and inhibition of long-term potentiation, altogether leading to impaired neuronal function, reduced cognition and a decline in memory [12–16]. The conversion between the different Aβ assembly states is dynamic and reversible [13, 14, 17–22]. Recent research gave rise to the fact that AD might be a prion-like disease with misfolded Aβ molecules acting as nucleation seeds and initiating aggregate formation by recruiting additional unfolded or oligomeric Aβ peptides and thereby accelerating amyloid growth [23, 24].

From the current point of view, elimination of Aβ oligomers may be the most promising therapeutic intervention to AD. Our approach was to identify therapeutically interesting compounds by selection of ligands, which modulate the dynamic equilibrium of Aβ_1–42_ species towards decomposition of toxic oligomers by binding of the ligand to monomeric Aβ and stabilizing it within the equilibrium and thereby shifting the equilibrium away from Aβ oligomers.

Phage display selection is suitable to identify small peptide ligand that bind to any target, for example, Aβ. The variation of phage display using the exact mirror image of the original target is called mirror image phage display. This method allows the identification of peptides that consist solely of D-enantiomeric amino acid residues [25, 26]. Such D-peptides are more protease-resistant than peptides consisting of L-enantiomeric amino acid residues and have a prolonged half-life in the body [27–30]. Additionally, D-peptides are assumed to show lower immunogenicity even though immunogenicity levels may depend on the dose and frequency of administration [27–29, 31, 32].

Previously, we have shown that the D-peptide D3, which was derived from a mirror image phage display selection, is able to reduce the amyloid plaque load in transgenic mice, improve cognition and reduce inflammation after oral application [33, 34]. This has proven the suitability of mirror image phage display to identify highly active compounds that are stable enough for oral administration.

Here, we set out to repeat this successful strategy, but with an important modification to yield small, D-enantiomeric peptides that show increased specificity for Aβ monomers in order to stabilize them. Based on a previously described combination of selection and counterselection [35] we used D-enantiomeric Aβ_1–42_ monomers as bait and added Aβ_1–42_ oligomers and fibrils as counterselective agents to achieve our goal.

## Materials and Methods

### Peptides

Mosd1 (ysyltsyhmwvr-NH_2_, all amino acids are D-enantiomers) with > 98% purity was purchased from peptides & elephants (Potsdam, Germany). N-terminally biotinylated and non-biotinylated D-enantiomeric Aβ_1–42_ was purchased from JPT (Berlin, Germany), N-terminal biotinylated L-enantiomeric Aβ_1–42_ was purchased from AnaSpec (Fremont, CA, USA) and L-enantiomeric Aβ_1–42_ was acquired from Bachem (Bubendorf, Switzerland).

### Preparation of seedless Aβ_1–42_ stock solutions

Generally, all Aβ_1–42_ preparations were handled in siliconized reaction tubes with protein low binding features (Protein LoBind tubes, Eppendorf AG, Hamburg, Germany) and prepared as seedless stock solutions as follows. Aβ_1–42_ was dissolved in PTFE-filtered 1,1,1,3,3,3-hexafluor-2-propanol (HFIP) and incubated at room temperature (RT) overnight. The HFIP was evaporated in a vacuum centrifuge at RT and additionally overnight in the open reaction tube covered with a lint-free wipe. The Aβ_1–42_ film was dissolved in filtered HFIP to 1 mM, aliquoted, sealed with parafilm and stored at -20°C. Prior to use, HFIP was evaporated.

### Separation of Aβ_1–42_ species via size exclusion chromatography (SEC)

In order to obtain monomeric and oligomeric Aβ_1–42_ fractions, size exclusion gel filtration chromatography was executed following an optimized protocol of Johansson *et al*. [36]. Seedless Aβ_1–42_ in HFIP was dried in a vacuum centrifuge and solved in elution buffer (50 mM sodium phosphate, 150 mM NaCl, pH 7.4) to a concentration of 250 μM. After one minute vortexing, the sample was sonicated for one minute and centrifuged for 60 seconds at 16,000 x g to precipitate eventually undissolved remains. The supernatant was transferred in a syringe to a Superdex 75 10/300 GL column connected to an ÄKTA purifier FPLC chromatography system (both GE Healthcare Europe GmbH, Freiburg, Germany). Size exclusion was executed at a flow rate of 0.6 ml per minute and detection took place at 214 nm. The desired fractions were collected and pooled. Aβ_1–42_ oligomers eluted at about 8 ml and Aβ_1–42_ monomers at approximately 14 ml.

### Preparation of Aβ_1–42_ samples with a broad range of different sized species

In order to gain a solution of Aβ_1–42_ which resembles several toxic and nontoxic Aβ species over a broad size range, 80 μM seedless Aβ_1–42_ was incubated in 10 mM sodium phosphate buffer (pH 7.4) for 4.5 hours at RT and shaking at 600 rpm.

### Preparation of Aβ_1–42_ fibrils

In order to gain Aβ_1–42_ fibrils, seedless Aβ_1–42_ was incubated at 80 μM in 10 mM sodium phosphate buffer (pH 7.4) at RT for at least 24 hours with shaking at 600 rpm. To separate the fibrils from other species, the sample was loaded onto an iodixanol density gradient as described below. Fractions containing fibrils and high molecular weight (HMW) aggregates (fractions 12–14) were used for further experiments.

### Density gradient centrifugation (DGC)

In order to separate differently sized Aβ_1–42_ assemblies, 100 μl of the sample were added on the top of a discontinuous gradient of iodixanol (OptiPrep, AXIS-SHIELD, Oslo, Norway). The gradient was prepared by layering 260 μl of 50% iodixanol at the bottom of a 11 x 34 mm polyallomer centrifuge tube, overlaid by 260 μl of 40%, 260 μl of 30%, 780 μl of 20%, 260 μl of 10% and 100 μl of 5% iodixanol. The samples were spun at 260,000 x g for three hours at 4°C in an Optima MAX-XP ultracentrifuge with a TLS-55 rotor (both Beckman Instruments, Brea, USA). After centrifugation, 14 fractions of 140 μl each were harvested with a pipette from top to bottom.

### Competitive mirror image phage display

In order to obtain specifically Aβ_1–42_ monomer binding species, a mirror image phage display with six panning rounds was performed. To reduce binding of phages to larger Aβ_1–42_ species, Aβ_1–42_ oligomers and fibrils were added, starting from round two, as counter-selective agents. Phages binding to Aβ_1–42_ oligomers and fibrils were subsequently removed from the solution leading to an enrichment of phages, which exclusively bind monomeric Aβ_1–42_.

The target peptide, SEC-derived monomeric, D-enantiomeric, N-terminally biotinylated Aβ_1–42_, was immobilized to plates with alternating surface properties. In order to avoid selection of plastic binding phages, different plastic surfaces were used in each subsequent round of panning (Nunc 96-well Immobilizer Streptavidin microwell plate, polystyrene (Thermo Fisher Scientific Inc., Waltham, MA, USA), polypropylene and polycarbonate microwell plates 96-well (BioTeZ Berlin Buch GmbH, Berlin, Germany)). According to the manufacturer, the polystyrene plates were pretreated by washing three times with 300 μl of 1x TBS containing 0.05% (v/v) Tween-20 per well. Additionally, in every second round, the surface was blocked with 150 μl of 1x TBS / 0.1% (v/v) Tween-20 / 1% BSA (w/v) per well with gentle shaking for one hour at RT prior to the addition of the target peptide to reduce unspecific binding. Thus, no combination of plate surface and blocking was used twice during the six panning rounds performed.

One-hundred microliters of a 63 nM solution of target peptide diluted in 1x TBS per well was immobilized to the well for five minutes at RT, followed by three washing steps with 150 μl of 1x TBS.

In the first panning round 90 μl of 1x TBS were mixed with 10 μl of the Ph.D.-12 Phage Display Peptide Library (New England Biolabs GmbH, Frankfurt/M, Germany) and added to the well for five minutes of incubation at RT. The solution was removed and 100 μl of 10 μM biotin in 1x TBS / 0.1% (v/v) Tween-20 (TBS-T) were added for five minutes incubation at RT to reduce streptavidin-binding phages. Subsequently, the well was washed four times with TBS-T.

Elution of bound phages was conducted by adding 100 μl of 0.2 M glycine-HCl (pH 2.2) for ten minutes at RT. The solution was removed and subsequently added to a fresh reaction tube with 25 μl of 1 M Tris-HCl (pH 9.1) in order to neutralize the solution. Twenty microliters of the solution were then used for phage titer determination after elution (output titer). The remaining volume was used for amplification of the eluted phages.

Determination of the output titer and phage amplification was conducted according to the distributor´s manual. Shortly, 100 μl of phage dilutions ranging from 10^−2^ to 10^−7^ were mixed with 100 μl *E*. *coli* K12 ER2738 cells at an optical density of 0.6. The mixture was plated together with 800 μl top agar per dilution on LB/Tet/IPTG/XGal Petri dishes (40 x 10 mm) and incubated overnight at 37°C. The next day, plaques were counted and the titer was determined.

For phage amplification, 20 ml *E*. *coli* K12 ER2738 cell solution were grown to an optical density of 0.1 and incubated with the remaining volume of eluted phages (105 μl) for 4.5 hours at 37°C and 160 rpm. After incubation, the culture was centrifuged for 20 minutes at 2,300 x g at 4°C and 1 ml of the supernatant was removed and stored at 4°C. The remaining volume was precipitated overnight at 4°C with 7 ml PEG-8000 / 2.5 M NaCl. Subsequently, the solution was centrifuged for 60 minutes at 3,000 x g and 4°C whereupon the supernatant was discarded. After dissolving the pellet in 1 ml of 1x TBS, the sample was centrifuged for five minutes at 9,300 x g at 4°C. The supernatant was mixed with 200 μl PEG-8000/2.5 M NaCl, followed by 60 minutes incubation on ice and a final centrifugation step for 20 minutes at 16,000 x g and 4°C. The supernatant was removed and the pellet was resuspended in 100 μl of 1x TBS. The input titer was measured analogically to the output titration with dilutions ranging from 10^−8^ to 10^−13^.

While the concentration of the target peptide remained stable, D-enantiomeric Aβ_1–42_ oligomers and high molecular weight (HMW) aggregates/fibrils without an N-terminal biotin tag were added, starting from panning round two in increasing concentrations (round 2: 1 nM—round 3: 5 nM—round 4: 10 nM—round 5: 50 nM—round 6: 500 nM) as will be explained below. Aβ_1–42_ oligomers and HMW aggregates and fibrils were obtained from SEC and DGC, respectively, as described above. In order to obtain Aβ_1–42_ HMW aggregates and fibrils fractions 12, 13 and 14 from the DGC were combined. Due to the addition of the competition step with Aβ_1–42_ oligomers and HMW aggregates/, the protocol was adjusted starting from the second panning round as follows. The amplified phages from the previous round were diluted to 1x10^11^ phages in 60 μl of 1x TBS and added to the well previously coated with SEC-derived, D-enantiomeric, N-terminally biotinylated Aβ_1–42_ monomers. Twenty microliters of SEC-derived Aβ_1–42_ oligomers and 20 μl of DGC-separated Aβ_1–42_ HMW / fibrils, each diluted in 1x TBS to the above mentioned concentration (e.g. for the second panning round 1 nM and 5 nM for panning round 3), were additionally added to the sample. After five minutes of incubation at RT, the solution was removed and the procedure went on with the aforementioned biotin competition step. The amount of washing steps increased with every panning round (4–6–8–10–12–15).

### Single phage amplification

Single plaque forming units, each representing phages grown from one single clone, were picked from the output titer plates from round three to six and added to 5 ml of a *E*.*coli* K12 ER2738 culture at an optical density of 0.1 grown in LB/tetracycline medium and were then amplified for 4.5 h hours at 37°C at 160 rpm. Cultures were centrifuged for 20 minutes at 3,000 x g and 4°C and the supernatant was removed. Two milliliters were used for DNA extraction and kept at 4°C until usage, one milliliter was aliquoted for single phage ELISA and 0.5 ml were mixed with 0.5 ml 80% sterile glycerin as backup and stored at -80°C.

### Single phage ELISA

The specificity and affinity of single phage clones towards the target peptide and the counterselective agents Aβ_1–42_ oligomers and HMW aggregates / fibrils was analyzed by ELISA.

320 nM of SEC-derived N-terminally biotinylated D-Aβ_1–42_ monomers or 320 nM SEC-derived N-terminally biotinylated D-Aβ_1–42_ oligomers mixed with DGC-derived N-terminally biotinylated Aβ_1–42_ HMW aggregates/fibrils were immobilized in duplicates for each single phage clone. Therefore, the total amount of Aβ_1–42_ in each well was 150 ng. Amplified single phage clones from panning rounds three to six were analyzed. Additionally, the different species were immobilized to two wells each in order to check the immobilization efficiency by the Aβ-specific antibody 6E10 (Beta Amyloid 1–16 (6E10) monoclonal antibody, BioLegend, Dedham, MA, USA). Non-coated wells were used as additional controls for each phage. Additionally, buffer instead of phages was applied as control for cross reactivity of the anti M13:HRP conjugated antibody to the well surface and the immobilized target in duplicate.

The microtiter plates (Nunc 96-well Immobilizer Streptavidin microwell plate, Thermo Fisher Scientific Inc., Waltham, MA, USA) were pretreated as recommended by the manufacturer. The aforementioned Aβ_1–42_ species were diluted in 1x TBS to 320 nM each and 100 μl per well were incubated for 15 minutes at RT with gentle shaking. After washing the wells twice with 150 μl of 1x TBS and blocking for one hour at RT with 1% (w/v) BSA in TBS-T, the plates were washed again three times with 150 μl of TBS-T. During the blocking step, the amplified phages and the buffer control (LB medium) were mixed with 1% (w/v) BSA in TBS-T in a ratio of 1:1 and incubated on a shaker at RT for 20 minutes. Afterwards 100 μl of the phage suspensions, the buffer control and the first antibody solution (6E10 1:1,000 in TBS-T) were given to the wells for one hour with gentle shaking at RT. After washing the plates for five times with 150 μl of TBS-T, 200 μl of TBS-T were added to each well and the plates were incubated at RT for another hour.

The supernatant was removed and 100 μl of the antibody dilutions were added to the adequate wells for one hour at RT (mouse anti bacteriophage M13 major coat protein (p8) HRP conjugated, GE Healthcare Europe GmbH, Freiburg, Germany and goat anti mouse IgG (H+L) HRP conjugated, Thermo Fisher Scientific Inc., Rockford, IL, USA, respectively). The anti-M13 antibody was diluted 1:5,000 in TBS-T and the goat anti mouse IgG antibody was diluted 1:1,000 in the same buffer. Subsequently, the plates were washed ten times with 150 μl of TBS-T and detection was conducted by measuring the conversion of the substrate 3,3',5,5'-tetramethylbenzidine (TMB) by HRP accompanied with a color change. Therefore, 50 μl of a TMB solution (one pill TMB was dissolved in 1 ml DMSO and diluted with 9 ml sterile filtered 0.05 M phosphate citrate buffer) was added to each well and color change was stopped by adding 50 μl of 2 M H_2_SO_4_ when the solution in the wells started getting turquoise. The absorption at 450 nm was measured in a microplate reader. After subtraction of the buffer control, the absorption at 450 nm for the wells coated with Aβ_1–42_ oligomers and fibrils were normalized to the Aβ_1–42_ content of the Aβ_1–42_ monomer-coated wells as determined by 6E10.

### DNA extraction for sequencing

The single stranded phage DNA was purified as described in the NEB phage display manual (Instruction manual, version 2.7) with the exception that the iodide buffer was exchanged by a 10:1 mixture of 3M sodium acetate (pH 5.2) and TE buffer. Sequencing was conducted by GATC Biotech AG (Konstanz, Germany).

### Transmission electron microscopy (TEM)

In order to analyze which Aβ_1–42_ species have formed after incubation with the peptide Mosd1, TEM images were taken. Ten micromolar seedless Aβ_1–42_ was incubated with or without 10 μM Mosd1 for 24 hours at RT. Twenty microliters of each sample were spotted on a formvar/carbon coated copper grid (Plano GmbH, Wetzlar, Germany) for three minutes. Afterwards the solution was detached with filter paper and the grids were washed three times with 20 μl of ddH_2_O and once with 5 μl 1% aqueous uranyl acetate. Then, 5 μl of the 1% uranyl acetate solution was applied to the grid for one minute for negative staining. The solution was removed and grids were dried overnight. The samples were analyzed with a Libra 120 transmission electron microscope (Carl Zeiss AG, Oberkochen, Germany) operating at 120 kV.

### Quantitative determination of interference with Aβ_1–42_ aggregate size distribution

The density gradient centrifugation method allows matrix-free separation and fractionation of different Aβ_1–42_ species according to their sedimentation coefficients which depend on size and shape.

Aβ_1–42_ was incubated as mentioned above (250 μl 80 μM seedless Aβ_1–42_ in 10 mM sodium phosphate buffer at pH 7.4, 4.5 hours, 600 rpm, RT) in order to gain a mixture of differently sized species. In order to analyze the influence of Mosd1, coincubation for 40 minutes at RT with different concentrations of the peptide (0-10-20-40-80 μM) followed.

One-hundred microliters were given on the top of a discontinuous iodixanol gradient and spun in an ultracentrifuge as described above. Fourteen fractions of 140 μl each were harvested from top to bottom after centrifugation and the residual pellet (60 μl) was mixed with 60 μl of 6 M guanidine hydrochloride and boiled for ten minutes. This sample represents the 15^th^ fraction. The samples were analyzed by RP-HPLC and Tris-tricine SDS-PAGE followed by silver staining.

Peptides were quantified via isocratic reversed-phase high performance liquid chromatography (RP-HPLC). The column used was a Zorbax SB-300-C8 on a 1260 Infinity system (both Agilent Technologies Deutschland GmbH, Böblingen, Germany). Twenty microliters of each sample were injected and run with 1 ml per minute in an aqueous 30% (v/v) acetonitrile / 0.1% (v/v) trifluoric acid buffer as mobile phase and 80°C column temperature to denature Aβ species and separate them from other components, especially iodixanol. The signal was detected at an absorbance of 214 nm. The data were recorded and peaks were integrated using the ChemStation software (Agilent Technologies). In order to calibrate the column, Aβ_1–42_ solutions of known concentration were used to plot peak area versus Aβ_1–42_ concentration. The plot equation then allowed calculation of Aβ_1–42_ concentration within the samples.

### Seeding assay (ThT)

In order to analyze the influence of Mosd1 on seeded growth of Aβ_1–42_, the seeding potential of fibrillary Aβ_1–42_ seeds on seedless Aβ_1–42_ was monitored via Thioflavin T (ThT) fluorescence.

Seedless Aβ_1–42_ (200 μM in 10 mM sodium phosphate buffer) were incubated at 37°C and 600 rpm for three days in order to gain seeding-competent Aβ_1–42_ fibrils. The sample was centrifuged at 14,000 x g for 45 minutes at 4°C in order to sediment fibrillary Aβ_1–42_ content. The supernatant was removed and the remaining Aβ_1–42_ fibrils were diluted in 100 μl 10 mM sodium phosphate buffer. After sonication for two minutes, concentration was determined by RP-HPLC. Aβ_1–42_ seeds were incubated with or without a fivefold molar excess of Mosd1 for additional 24 hours. The mixture of Aβ_1–42_ seeds and Mosd1 was again sonicated for two minutes and added to freshly prepared seedless Aβ_1–42_ (15 μM) and 20 μM ThT. The final concentrations were 1.88 μM Aβ_1–42_ seeds and 9.4 μM Mosd1. Each sample was added to a 96-well microtiter plate in triplicate and progression of fluorescence values was monitored over time at 24°C. The experiment was conducted independently for three times.

For each sample an asymmetric five parameter fit was used in order to determine the amplitude of relative fluorescence units and the half-life t_1/2_. After checking for Gaussian distribution by D´Agostino-Pearson omnibus normal test, significance was determined by one-way ANOVA.

### Cell viability assay (MTT reduction)

PC-12 cells (DSMZ-Deutsche Sammlung von Mikroorganismen und Zellkulturen GmbH, Braunschweig, Germany) were cultured in DMEM medium supplemented with 10% fetal calf serum, 1% antibiotics (Penicillin / Streptomycin) (all Sigma-Aldrich Corp., St.Louis, MO, USA) and 5% horse serum (PAA Laboratories GmbH, Pasching, Germany) on collagen A-coated (Biochrom GmbH, Berlin, Germany) tissue culture flasks (SPL Life Sciences Co., Korea) in a humidified incubator with 5% CO_2_ at 37°C and grown for a maximum of 15 passages. Medium was changed every two days and cells were passaged, according to their confluence, every three to five days.

PC-12 cells were seeded in clear, collagen-coated 96-well flat bottom microwell plates (Life Technologies Inc., Carlsbad, CA, USA) at a density of 1x10^4^ cells in a volume of 100 μl per well and incubated for 24 hours. Aβ_1–42_ / Mosd1 mixtures were incubated as already described in the above mentioned assay for quantitative determination of interference with Aβ_1–42_ aggregate size distribution. Mosd1 concentrations of 0/40/80 μM were used. From each well that contained cells 1.25 μl medium were removed and replaced with 1.25 μl of the preparations in order to gain a final concentration of 1 μM Aβ_1–42_ and 0/1/0.5 μM Mosd1 in every well, respectively. Every approach was tested three times at least in quintuplicates. The plates were incubated for 24 hours in the incubator with 5% CO_2_ at 37°C. Subsequently, 10 μl of the supernatant were taken out of every well and 10 μl of MTT reagent (Cell proliferation kit 1, Roche Diagnostics GmbH, Mannheim, Germany) were added for four hours of incubation in the incubator. Afterwards, 100 μl of solubilization reagent (Roche Diagnostics GmbH, Mannheim, Germany) were added to all wells and the plate was incubated overnight. The plate was shaken on a table shaker for five minutes to distribute the purple colored solution equally within each well and absorbance was measured at 660 nm and 570 nm with a microplate reader (POLARstar OPTIMA, BMG Labtech GmbH, Ortenberg, Germany). Reference values at 660 nm were subtracted from values for formazan absorbance (570 nm). The mean value of cell-free wells was subtracted as background. The arithmetic mean of all measurements per approach was calculated. Results are represented as the percentage of MTT reduction, assuming that the absorbance of control cells was 100%. Cells treated with 0.1% TritonX-100 in 10 mM sodium phosphate buffer (pH 7.4) served as control for dead cells. After analysis for Gaussian distribution by Shapiro-Wilk test, the values were tested for significance using the Mann-Whitney *U* test.

### Western blot to investigate γ-secretase inhibition

Neuro-2a cells (DSMZ, Braunschweig, Germany) and Neuro-2a cells stably transfected with human APP695 were cultured in DMEM with 10% fetal calf serum and 1x non-essential amino acids (both Sigma-Aldrich Corp., St.Louis, MO, USA) in tissue culture flasks in a humidified incubator with 5% CO_2_ at 37°C. Fifty thousand cells per well were seeded in 500 μl in a 24 well microwell plate for 24 hours at 37°C and 5% CO_2_ to assure attachment to the surface. A stock solution of 1 mM Mosd1 was prepared in sterile water and the γ-secretase inhibitor DAPT was diluted to 5 mM in DMSO, respectively. The dilutions were spun at 1,000 x g for one minute prior to use. Then 0/10/100 μM Mosd1 and 1 μM DAPT were added to the cell supernatant and DMSO as well as water were tested as control. The cells were incubated at 37°C and 5% CO_2_ for 24 hours. The cells were analyzed with a laser scanning microscope LSM 710 (Carl Zeiss AG, Oberkochen, Germany). Afterwards, the cells were washed three times with 1x PBS and lysed with 60 μl of NP-40 lysis buffer for 15 minutes. The lysates were resuspended, transferred to reaction tubes and centrifuged for five minutes at 12,000 x g. Afterwards, the supernatants were transferred to new reaction tubes and protein concentration was determined via microBCA assay.

For gel electrophoresis the lysates were adjusted to the lowest measured protein concentration. All samples were mixed with 4x Tris-tricine gel loading buffer (4% SDS (w/v), 12% Glycerin (v/v), 50 mM Tris, 2% β-mercaptoethanol (v/v), 0.01% SERVA BlueG (w/v); pH 6.8) in a ratio of 1:3 and boiled for ten minutes. Gel electrophoresis was conducted with 10% Tris-tricine gels at a constant voltage of 120 V. The PageRuler prestained protein ladder 10–170 kDa (Thermo Fisher Scientific, Inc., Rockford, IL, USA) served as marker.

After gel electrophoresis, the proteins were transferred via semi-dry blotting to a PVDF membrane. Protein transfer took place at 1 A and 25 V for 30 minutes. The membrane was blocked with 5% non-fat dry milk powder in 1x TBS-T at 4°C overnight with gentle shaking. Subsequently, the membrane was washed three times with TBS-T at RT and gentle shaking. The primary antibody for the detection of C-terminal fragment of APP (anti-amyloid precursor protein, C-terminal (751–770) rabbit pAb, Merck KGaA, Darmstadt, Germany) was diluted 1:5,000 in TBS-T. Simultaneously, the anti-β-actin antibody (β-actin (8H10D10) mouse mAb, Cell Signaling Technology, Inc., Danvers, MA, USA) was diluted 1:1,000 in TBS-T and both antibodies were given to the membrane, which was incubated overnight at 4°C on a roller mixer, subsequently. The membrane was washed three times with TBS-T and incubated with the secondary antibodies goat anti-rabbit IgG:HRP diluted 1:5,000 in TBS-T and goat anti-mouse IgG:HRP diluted 1:10,000 in TBS-T (both Santa Cruz Biotechnology, Inc., Dallas, Texas, USA) for three hours at RT on a roller mixer. After three washing steps with TBS-T, the membrane was incubated with the substrate for enhanced chemoluminescence (SuperSignal West Dura Chemoluminescent Substrate, Thermo Fisher Scientific, Inc., Rockford, IL, USA) for five minutes at RT in the dark. The signal was detected with the ChemiDoc MP gel documentation system (Bio-Rad Laboratories, Inc., Hercules, CA, USA).

## Results

N-terminally biotinylated, SEC-derived Aβ_1–42_ monomers were immobilized to streptavidin-coated microwell plates. To avoid selection of plastic binding ligands, different plastic surfaces were used in each round. In particular, polystyrene, polypropylene and polycarbonate served as surface, alternating every round. Additionally, in every second round, the surface was blocked with BSA. Due to this procedure, six different combinations of blocking and surface type were used during the panning rounds and not a single combination occurred twice. This step was important, because only very low concentrations of Aβ_1–42_ were immobilized in order to assure its monomeric state. Thus, there was a substantial risk that uncoated surface may have a strong bias during selection. In the first panning round, only Aβ_1–42_ monomers were offered to the naïve library of phages. For the next round, amplified phages from the previous round were added together with increasing concentrations of non-biotinylated counterselective Aβ_-42_ species (SEC-derived oligomers and DGC-derived HMW aggregates and fibrils). After panning, a biotin competition step was conducted to remove phages that compete with biotin. Afterwards the well was washed every round with increasing rigor and still bound phages were eluted and amplified.

**Fig 1:**
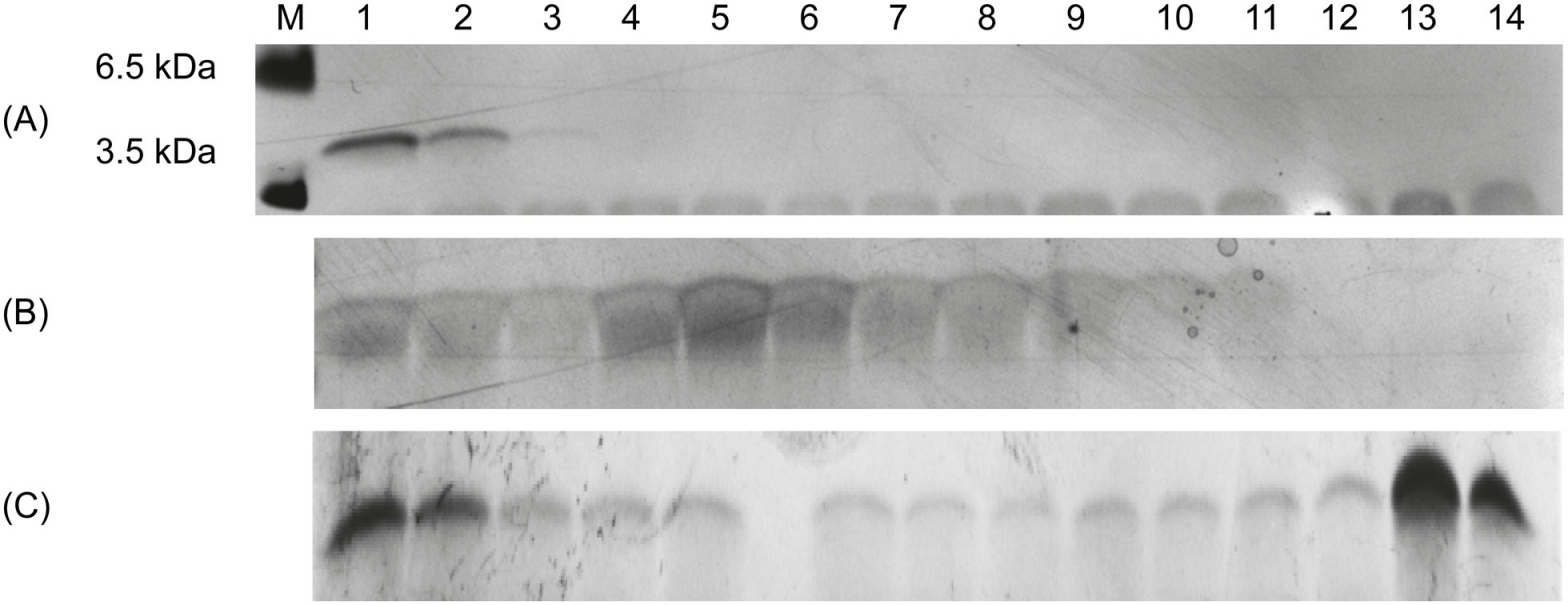
Silver stained Tris-Tricine-SDS-PAGE of different Aβ_1–42_ species. The Aβ_1–42_ species used for panning and counter selection during mirror image phage display were analyzed via DGC followed by Tris-Tricine-SDS-PAGE and silver staining. The SEC peak corresponding with Aβ_1–42_ monomers (A) presents Aβ_1–42_ content only in fractions 1 to 2 of a DGC gradient and therefore represents exclusively monomeric Aβ_1–42_. The SEC peak corresponding with Aβ_1–42_ oligomers (B) presents mainly Aβ_1–42_ in fractions 4 to 6, which is in accordance with oligomeric Aβ_1–42_ species. The preparation of fibrillary Aβ_1–42_ resulted in monomeric and fibrillary Aβ_1–42_ content as seen in fractions 1 to 2 and 13 to 14 (C). Using only the pooled fractions 12 to 14 ensured that no monomeric Aβ_1–42_ species were used as counterselective agent.

We verified the state of all used Aβ conformers by DGC followed by Tris-tricine-SDS-PAGE and silver staining. SEC-derived Aβ_1–42_ monomers, SEC-derived oligomers, and fibrillary Aβ_1–42_ were analyzed. As shown in Fig 1, prepared monomers are indeed free of any aggregates, as only the most upper fractions 1 and 2 of the DGC gradient contained Aβ. Oligomers can be found predominantly in fractions 4 to 6, and in smaller amounts also in neighboring fractions. Fibrils can be found in the extreme high molecular weight fractions, but obviously, during the DGC run, a substantial fraction of monomers dissociated from them and can be found in fractions 1 and 2.

Single phage clones from round three to six were amplified and sequenced. Sixteen sequences occurred more than once and were therefore tested for binding to monomeric Aβ_1–42_, Aβ_1–42_ oligomers and HMW aggregates / fibrils as well as non-coated wells in a single phage ELISA. Additionally, several clones with uniquely occurring sequences and four clones from a previously conducted mirror image phage display were tested. This former mirror image phage display was conducted with almost identical conditions. The experimental setup differed only in two details. Firstly, the counterselective Aβ_1–42_ HMW aggregates and fibrils were derived from an iodixanol gradient with different iodixanol concentrations (30–24–18–12–6 and 3% iodixanol instead of 50–40–30–20–10 and 5%). Secondly, the neutralization of the eluate took place in the same well as the elution step.

**Fig 2:**
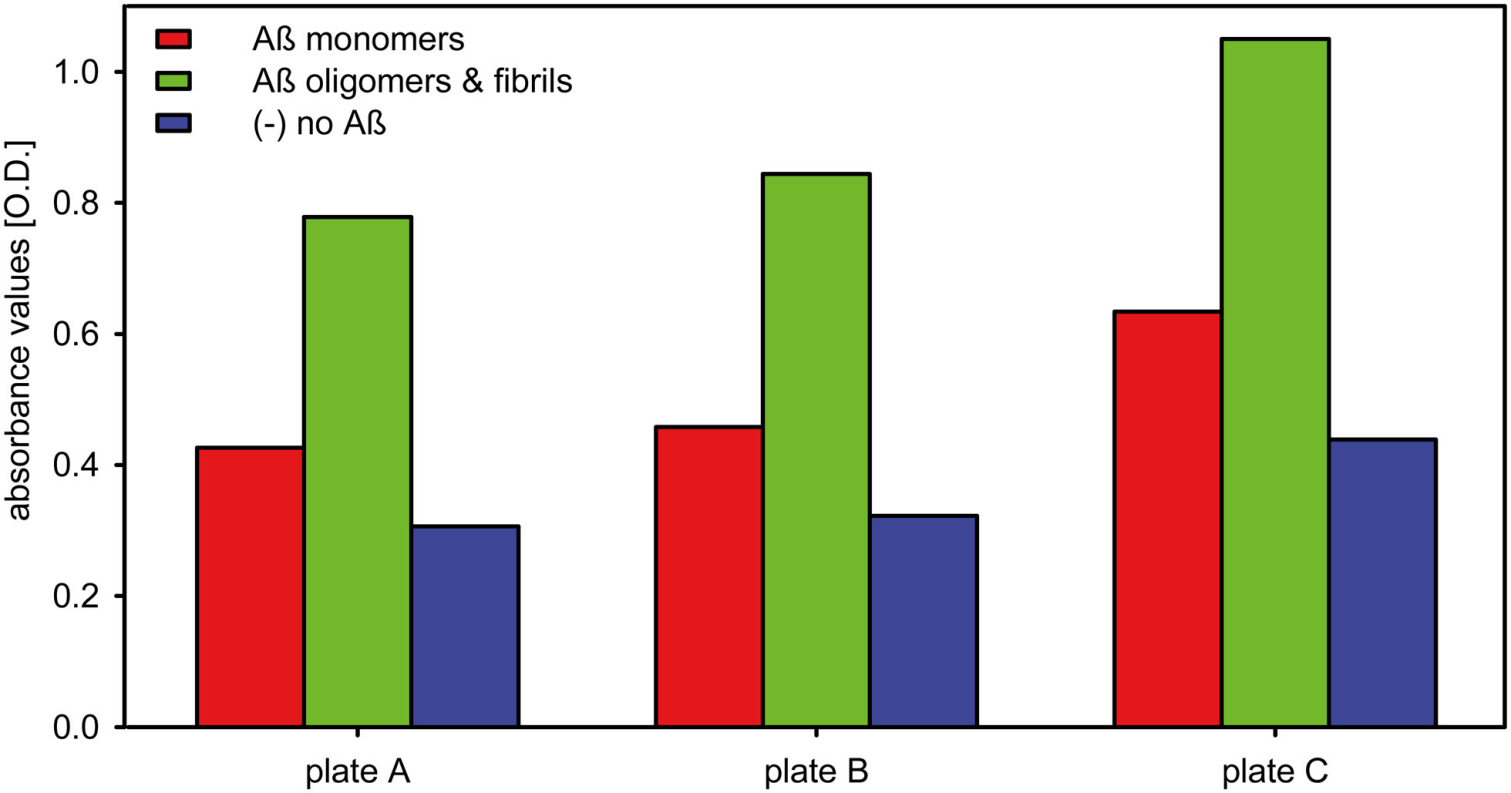
Single phage ELISA—immobilization control. The immobilization efficiency of different Aβ_1–42_ species was analyzed by the binding affinity of Aβ_1–42_ specific antibody 6E10 to immobilized Aβ_1–42_ on each plate used for single phage ELISA. The Aβ_1–42_ specific antibody 6E10 was added to wells coated with 150 ng Aβ_1–42_ monomers (red) or 150 ng Aβ_1–42_ oligomers and fibrils (1:1; green) or to wells only coated with streptavidin (blue). Transformation of substrate by the secondary antibody-conjugated HRP was measured at 450 nm.

**Fig 3:**
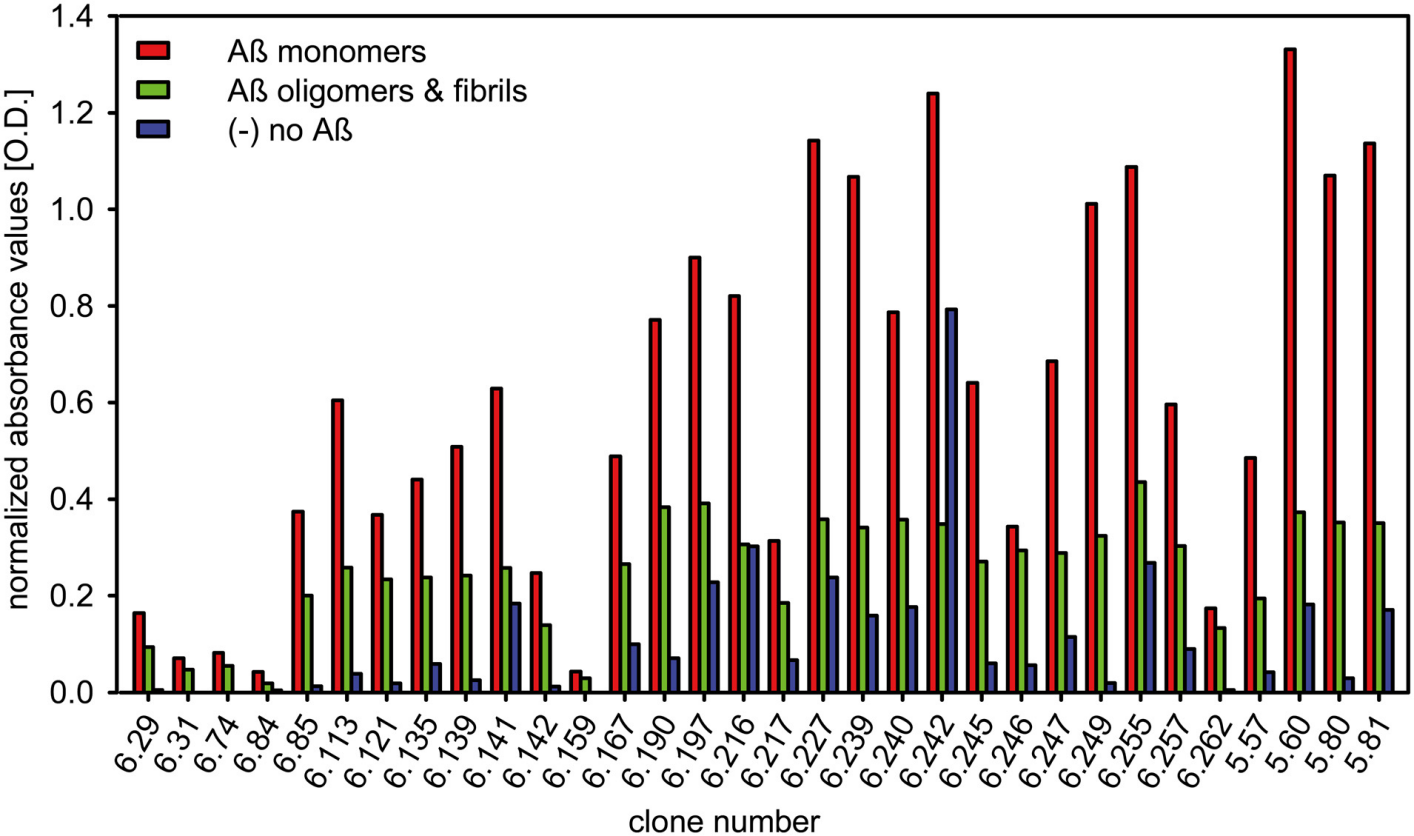
Single phage ELISA. The relative binding affinity of single phage clones from mirror image phage display (clone numbers 6.xx) and a previously conducted mirror image phage display (clone numbers 5.xx) to SEC-derived biotinylated Aβ_1–42_ monomers, oligomers and fibrils as well as the non-coated wells was analyzed. The M13 phage-specific antibody was used for detection. Transformation of substrate by the antibody-conjugated HRP was measured at 450 nm. Amplified single phage clones were added to wells coated with 150 ng Aβ_1–42_ monomers (red) or 150 ng Aβ_1–42_ oligomers and fibrils (1:1; green) or to wells only coated with streptavidin (blue). Cross reactivity of the M13 phage-specific antibody was tested in an approach without addition of phages. After background subtraction of the anti M13 antibody values, the values for phage to Aβ_1–42_ oligomer/fibril binding were normalized to the values of phage to Aβ_1–42_ monomer binding according to the outcome of coating efficiency controls with the Aβ specific antibody 6E10.

Fig 2 shows the immobilization efficiency of different Aβ_1–42_ species detected by Aβ_1–42_ specific antibody 6E10. On all plates used for single phage ELISA, Aβ_1–42_ monomers and oligomers/fibrils were immobilized in an amount which allowed normalization of the Aβ_1–42_ oligomer/fibril content to the content of Aβ_1–42_ in Aβ_1–42_ monomer-coated wells as determined by 6E10. As shown in Fig 3, the competitive mirror image phage display selection indeed yielded phages that display peptides that specifically bind to Aβ_1–42_ monomers as demonstrated by the strong binding to Aβ_1–42_ monomer coated wells and notably less binding to wells coated with Aβ_1–42_ oligomers and HMW aggregates or fibrils. We picked the clone with the highest signal intensity for monomeric Aβ_1–42_, but also the highest specificity for monomeric Aβ_1–42_ as deduced from the high ratio of binding to Aβ_1–42_ monomers and binding to oligomeric and fibrillary Aβ_1–42_. The amino acid sequence of the chosen clone 5.60 was YSYLTSYHMVWR. The corresponding D-enantiomeric peptide, named Mosd1 (monomer specific d-peptide 1), was synthesized from purely D-amino acids with its C-terminus amidated, and was further characterized.

**Fig 4:**
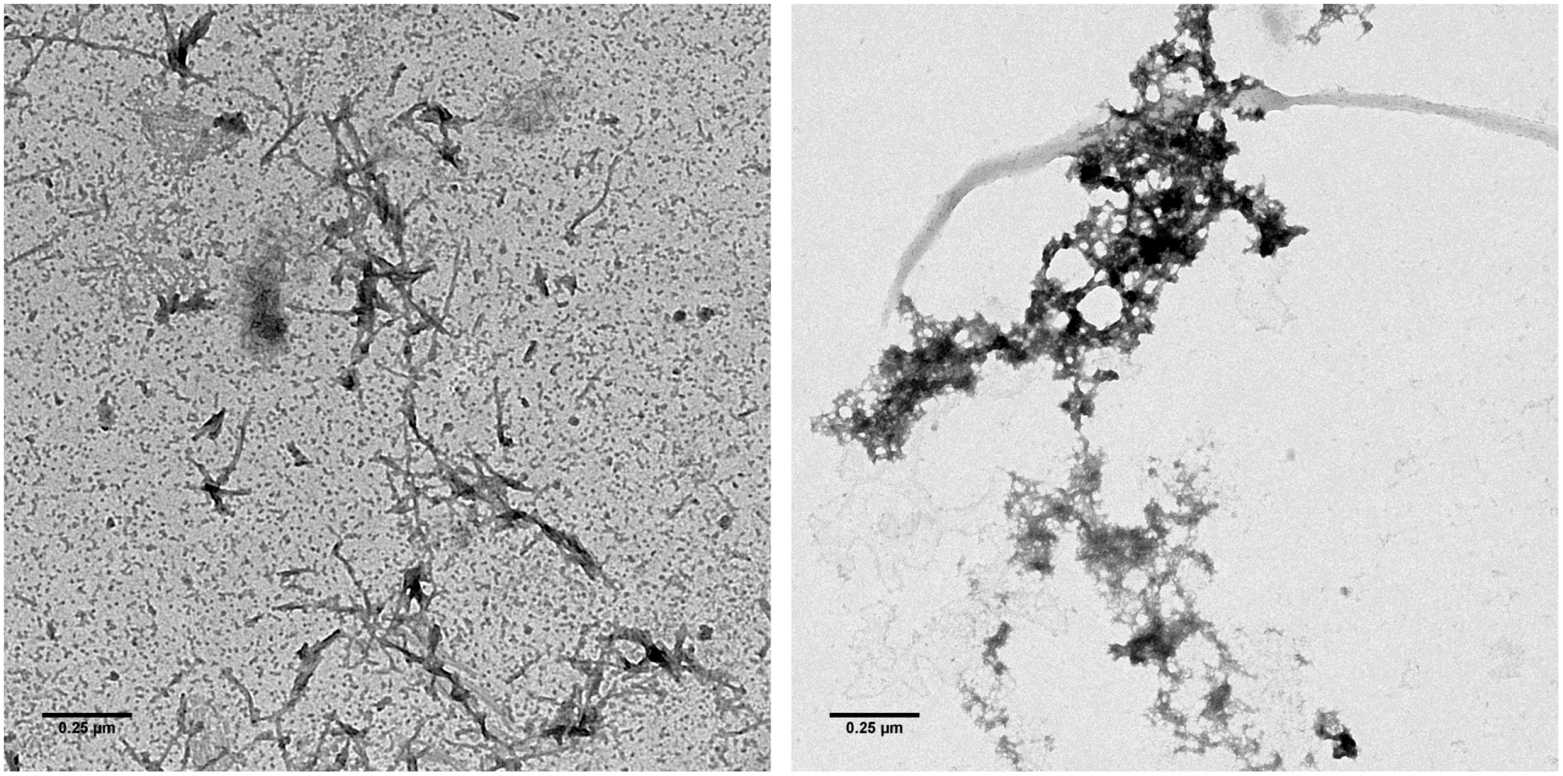
Transmission electron microscopy. After incubation of 10 μM pretreated Aβ_1–42_ without (left picture) and with 10 μM Mosd1 (right picture) for 24 hours at room temperature, samples were spotted onto a formvar/carbon coated copper grid and stained with 1% aqueous uranyl acetate. Samples were analyzed with a Libra 120 TEM operating at 120 kV. Scale bar presents 0.25 μm.

In order to obtain information on how Mosd1 changes Aβ_1–42_ aggregation, TEM pictures were taken after 24 hours incubation of Aβ_1–42_ with and without an equimolar ratio of Mosd1. The Aβ_1–42_ sample without Mosd1 contained a mesh of fibrils (Fig 4A). Additionally, Aβ oligomers and large aggregates (dot-like structures) were present, displaying a broad range of aggregated Aβ_1–42_ species. In the sample with Aβ_1–42_ and Mosd1 neither fibrils nor oligomers were observed. Instead, amorphous, unstructured aggregates were the major species found on the grid (Fig 4B).

After incubation of 80 μM Aβ_1–42_ followed by coincubation with different concentrations of Mosd1 (0/10/20/40/80 μM), samples were loaded on a discontinuous iodixanol gradient and centrifuged. Fifteen fractions were harvested from top to bottom, applied to Tris-tricine-SDS-PAGE and silver stained. With this experiment we were able to show that Mosd1 abolishes different Aβ_1–42_ species from the sample, which contains Aβ_1–42_ species of different size, ranging from monomers to fibrils and HMW aggregates.

**Fig 5:**
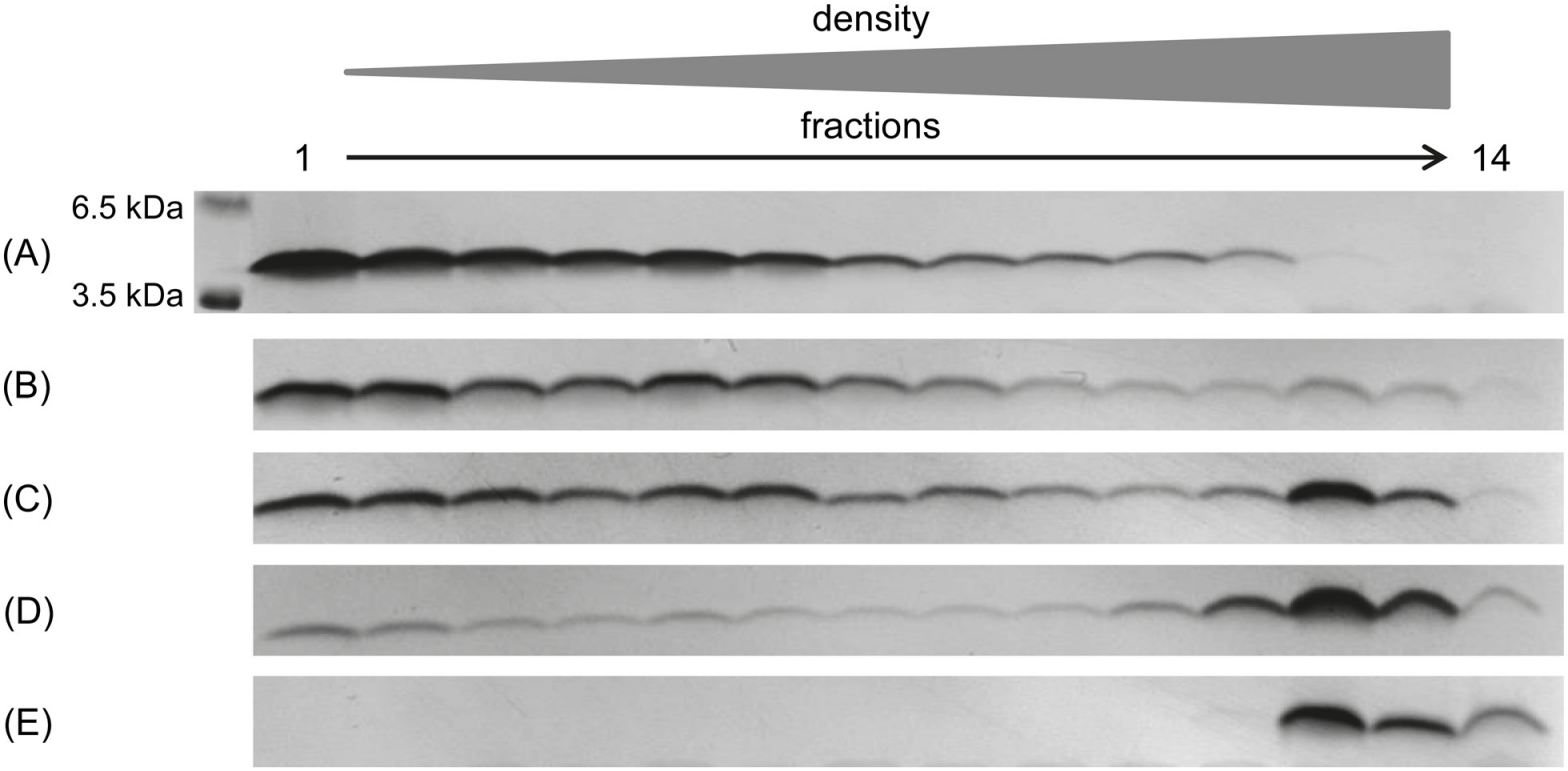
Qualitative Aβ_1–42_ distribution alteration. Silver staining of SDS gels after incubation of 80 μM Aβ_1–42_ for 4.5 hours at RT and 600 rpm and additional coincubation for 40 minutes with 0 (A) / 10 (B) / 20 (C) / 40 (D) / 80 μM (E) Mosd1 followed by density gradient centrifugation for three hours at 4°C at 259,000 x g. The bands display the signal for Aβ_1–42_ (4.5 kDa).

The distribution of 80 μM Aβ_1–42_, incubated for 4.5 hours without addition of Mosd1, showed a broad range of species distributed from fraction 1 to 12, representing monomers, small and large oligomers, protofibrils, fibrils and HMW aggregates (Fig 5A) [37]. The amount of Aβ_1–42_ as estimated from silver staining decreased with the fraction number, indicating substantial amounts of Aβ_1–42_ in form of monomers and small oligomers. The most toxic oligomers can be expected in fractions four to six [38]. The addition of Mosd1 shifted the distribution of Aβ_1–42_ towards large aggregates (Fig 5B–5E). This aggregates are suspected to be non-toxic [39]. The Aβ_1–42_ contents in fractions one to ten were decreased with increasing Mosd1 concentrations. We therefore conclude that Mosd1 decreased the amount of toxic Aβ_1–42_ oligomers and shifted the distribution equilibrium towards non-toxic aggregates in a concentration dependent manner.

**Fig 6:**
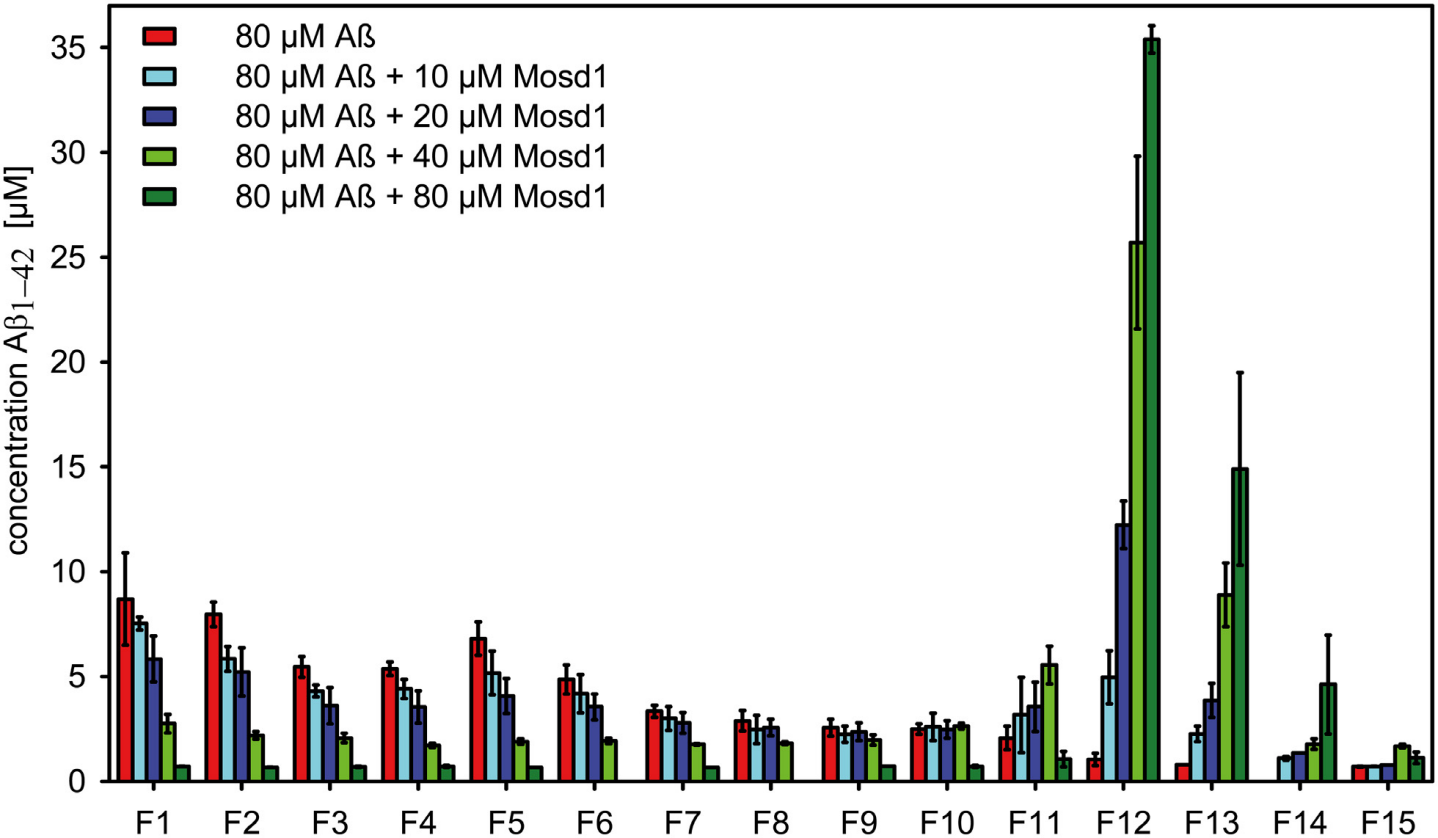
Quantitative concentration determination (RP-HPLC) of Aβ_1–42_ distribution. Concentrations of Aβ_1–42_ in each DGC fraction were determined quantitatively via RP-HPLC. Samples were loaded to a Zorbax 300SB-C8 column connected to a 1260 Infinity HPLC system. Separation of the samples was achieved by elevated column temperature (80°C) and an isocratic mobile phase of 30% acetonitrile/0.1% TFA in water. The averaged concentration of Aβ_1–42_ from three independent experiments (with standard deviation) is plotted against the obtained fractions F1 to F15 of different incubation approaches of Aβ_1–42_ without or with Mosd1. Shown in red are the concentrations of fractions from 80 μM Aβ_1–42_ incubated without Mosd1. The following columns represent the Aβ_1–42_ concentrations in the fractions from 80 μM Aβ_1–42_ samples coincubated with increasing concentrations of Mosd1 (10 μM = light blue; 20 μM = dark blue; 40 μM = light green; 80 μM = dark green).

Silver staining does not allow a quantitative determination of the detected species. In order to gain quantitative information about Aβ_1–42_ concentrations in every fraction, samples of each fraction were applied to RP-HPLC chromatography and analyzed for their Aβ_1–42_ contents, as described before [40]. The QIAD (quantitative determination of interference with Aβ_1–42_ aggregate size distribution) assay fully confirmed the above described behavior of Mosd1 and demonstrates that Aβ_1–42_ oligomer elimination by Mosd1 is concentration dependent (Fig 6). Most importantly, Mosd1 is able to almost completely eliminate Aβ_1–42_ oligomers.

**Fig 7:**
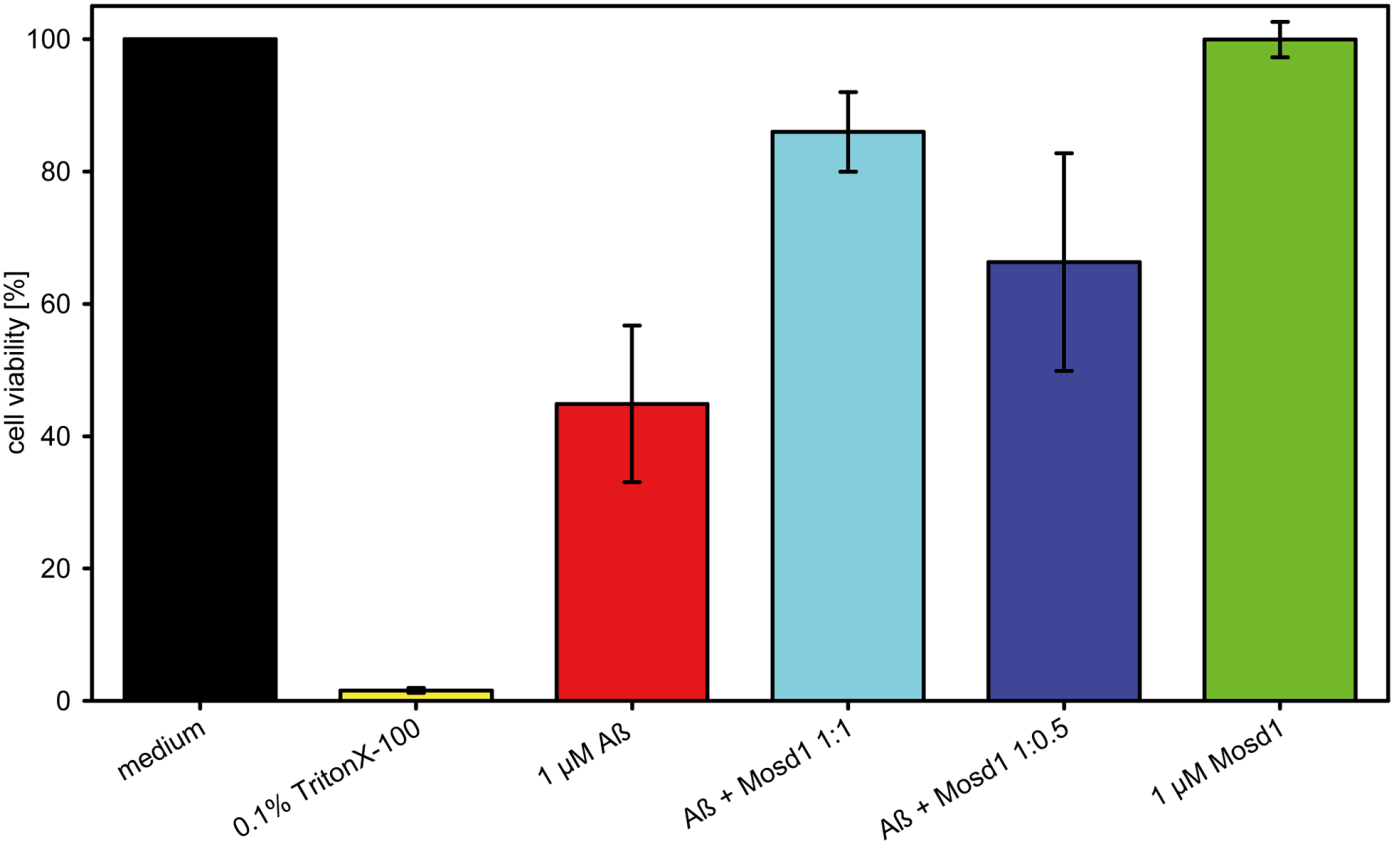
Reduction of seeded Aβ_1–42_ growth and fibrillar Aβ_1–42_ content. Seedless Aβ_1–42_ was incubated alone (black) or together with fibrillary Aβ_1–42_ seeds previously incubated with (green) or without (red) a fivefold molar excess of Mosd1. ThT (20 μM) was added to each sample in order to measure fibrillar content. The data were fitted with an asymmetric five parameter fit. The (A) amplitude of relative fluorescence (RFU) of fibrillated seedless Aβ_1–42_ served as 100% to which the other values were normalized. The (B) half-life (t_1/2_), displaying the point in time, when half of the maximum ThT signal (i.e. fibrillary content) was reached. Statistical significance was determined by one-way ANOVA. Error bars display SEM. ns: p > 0.05; *: p ≤ 0.05; **: p ≤ 0.01; ***: p ≤ 0.001.

A characteristic property of fibrillar Aβ to is its ability to act as a seed for conversion of monomeric Aβ into fibrillar Aβ assemblies. We therefore analyzed the impact of Mosd1 on fibril seeded fibrillation of Aβ_1–42_ using the Thioflavin T (ThT) assay (Fig 7). Fibrillar Aβ_1–42_ seeds were grown and afterwards incubated with or without Mosd1. ThT was added and the mixture was added to freshly prepared seedless Aβ_1–42_. The progression of ThT fluorescence was monitored over time. Compared to freshly prepared seedless Aβ_1–42_ (t_1/2_ = 19.69 h), addition of fibrillary Aβ_1–42_ seeds significantly reduced the time needed to increase fibrillar content (t_1/2_ = 0.57 h). When fibrillary Aβ_1–42_ seeds were coincubated with Mosd1 prior to addition of freshly prepared seedless Aβ_1–42_, the time for the increase of fibrillary content was significantly reduced as well, yet the time for the increase was significantly longer (t_1/2_ = 3.36 h) compared to the sample without Mosd1 (Fig 7A). Furthermore, the amount of fibrillar Aβ_1–42_ content in the samples, displayed by the amplitude of relative fluorescence units (Fig 7B), did differ significantly between seedless Aβ_1–42_ (set to 100% RFU) and seedless Aβ_1–42_ incubated with fibrillary Aβ_1–42_ seeds (84%) as well as between seedless Aβ_1–42_ and fibrillary Aβ_1–42_ seeds coincubated with Mosd1 (74%).

**Fig 8:**
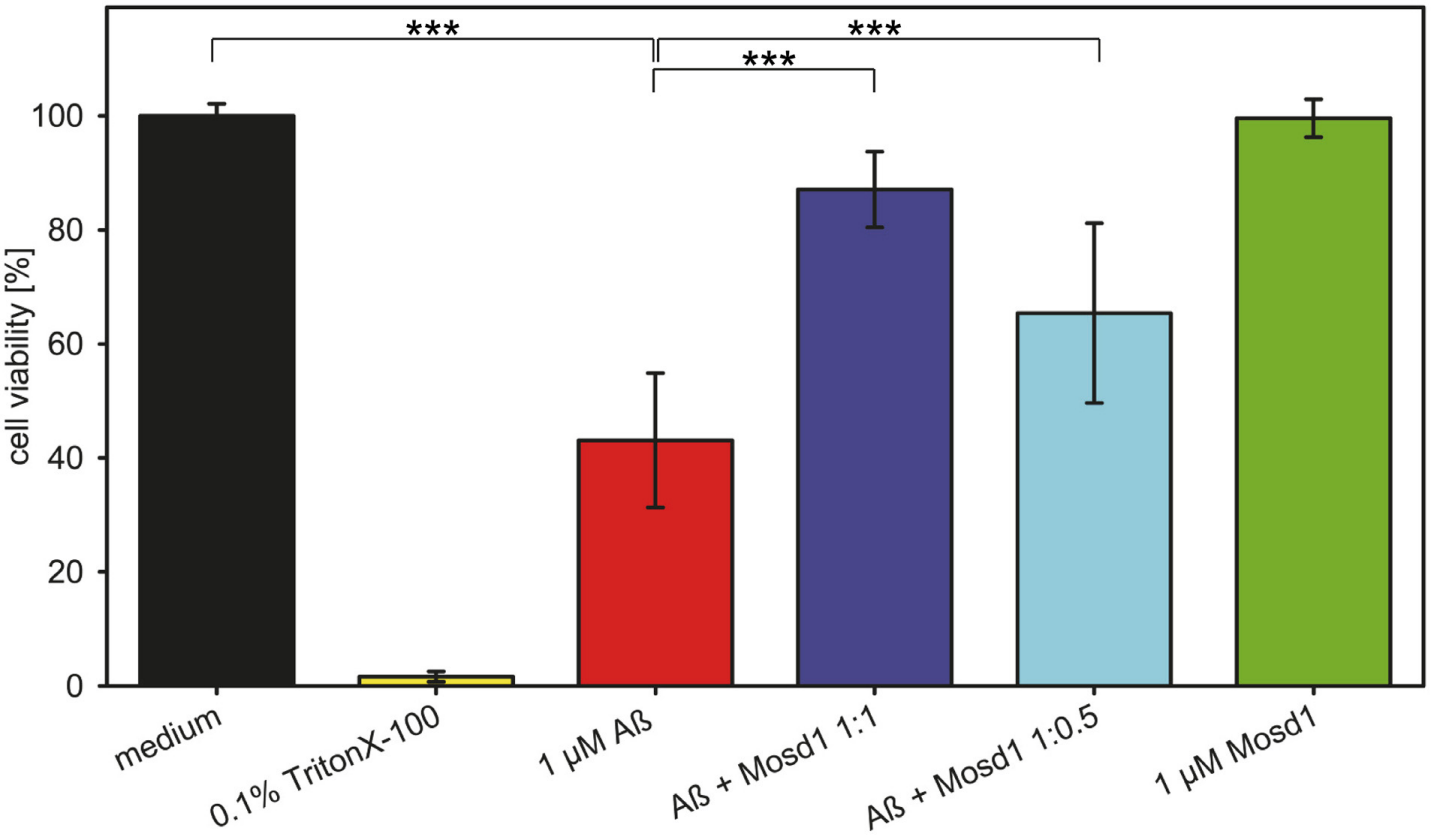
Effect of Aβ_1–42_ and Mosd1 on cell viability. Aβ_1–42_ and Mosd1 were tested for their influence on PC-12 cell viability by MTT reduction assay. Cell viability (in percent) is plotted against different treatment conditions. Adherently grown PC-12 cells were incubated 24 hours with medium (black) or 0.1% TritonX-100 (yellow) as controls for viable cells and cytotoxicity, respectively. Additionally, cells incubated for 24 hours with 1 μM Aβ_1–42_ (red), Aβ_1–42_ + Mosd1 1:1 (light blue) and 1:0.5 (dark blue), respectively. The green bar corresponds to cells incubated with 1 μM Mosd1 for 24 hours. Viability was analyzed by subsequent incubation with MTT substrate for four hours. After solubilization, absorbance was measured at 570 nm. The averages and standard deviations of absorbance values from five independently performed experiments were calculated and normalized to untreated cells (medium). Statistical significance was tested with Mann-Whitney *U* test. ***: p ≤ 0.001.

In order to confirm that Mosd1 yields Aβ_1–42_ species that are not toxic to cells and Mosd1 can indeed abolish the cytotoxic effect of Aβ_1–42_ and rescue cell viability, an MTT assay was performed (Fig 8). PC-12 cells were incubated with Aβ_1–42_ with or without different concentrations of Mosd1, derived from the same incubation method as mentioned above. Mosd1 without Aβ_1–42_ was tested to ensure that Mosd1 itself is not toxic to the cells. TritonX-100 was used as a control for cell death. Untreated cells were set 100% viable and all other values were normalized to this value. Incubation with 0.1% TritonX-100 resulted in 1.6% viable cells. The Aβ_1–42_ composition led to a significantly decreased cell viability of 45% and was therefore proven to be toxic. Mosd1 in a concentration of 1 μM did not alter MTT reduction in this assay and showed no toxic effect in PC-12 cells. When coincubated with Mosd1, the toxic effect of Aβ_1–42_ was significantly decreased with increasing concentrations of Mosd1. In an equimolar ratio, 86% of the cells stayed viable and with half the concentration of Mosd1 still 66% of the cells were viable. Mosd1 is therefore able to rescue PC-12 cells from Aβ_1–42_ induced toxicity.

**Fig 9:**
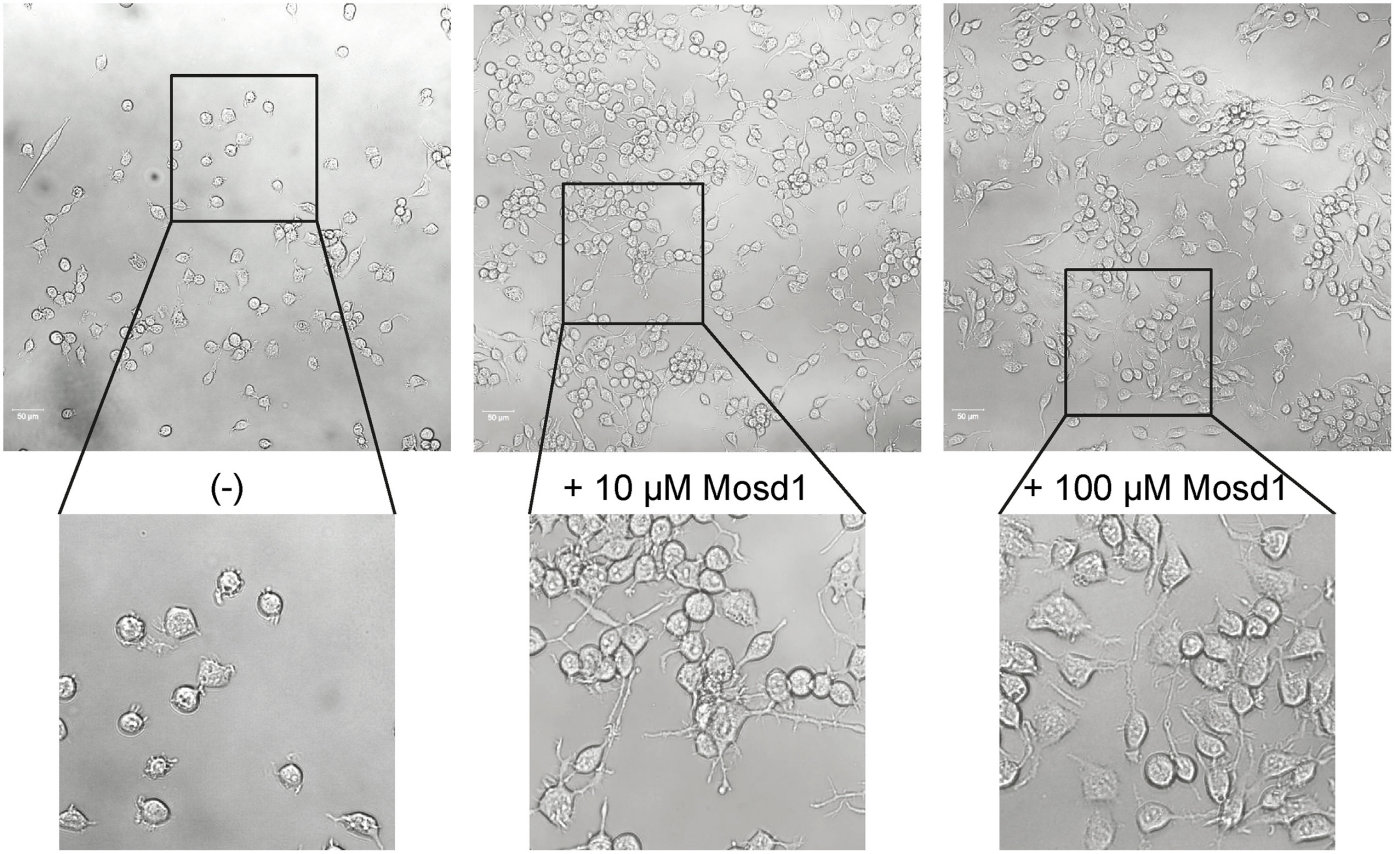
Effect of Mosd1 on Neuro-2a cells. Overview and detailed pictures of wild type Neuro-2a cells are shown. Neuro-2a cells were treated with 0, 10 and 100 μM of Mosd1, respectively. The left panel shows untreated wild type Neuro-2a cells. In the middle and right panel, incubation of wild type Neuro-2a cells with 10 μM Mosd1 and 100 μM Mosd1 are shown. Cell viability and morphology were analyzed with a LSM 710 laser scanning microscope. Scale bars equate 50 μm.

**Fig 10:**
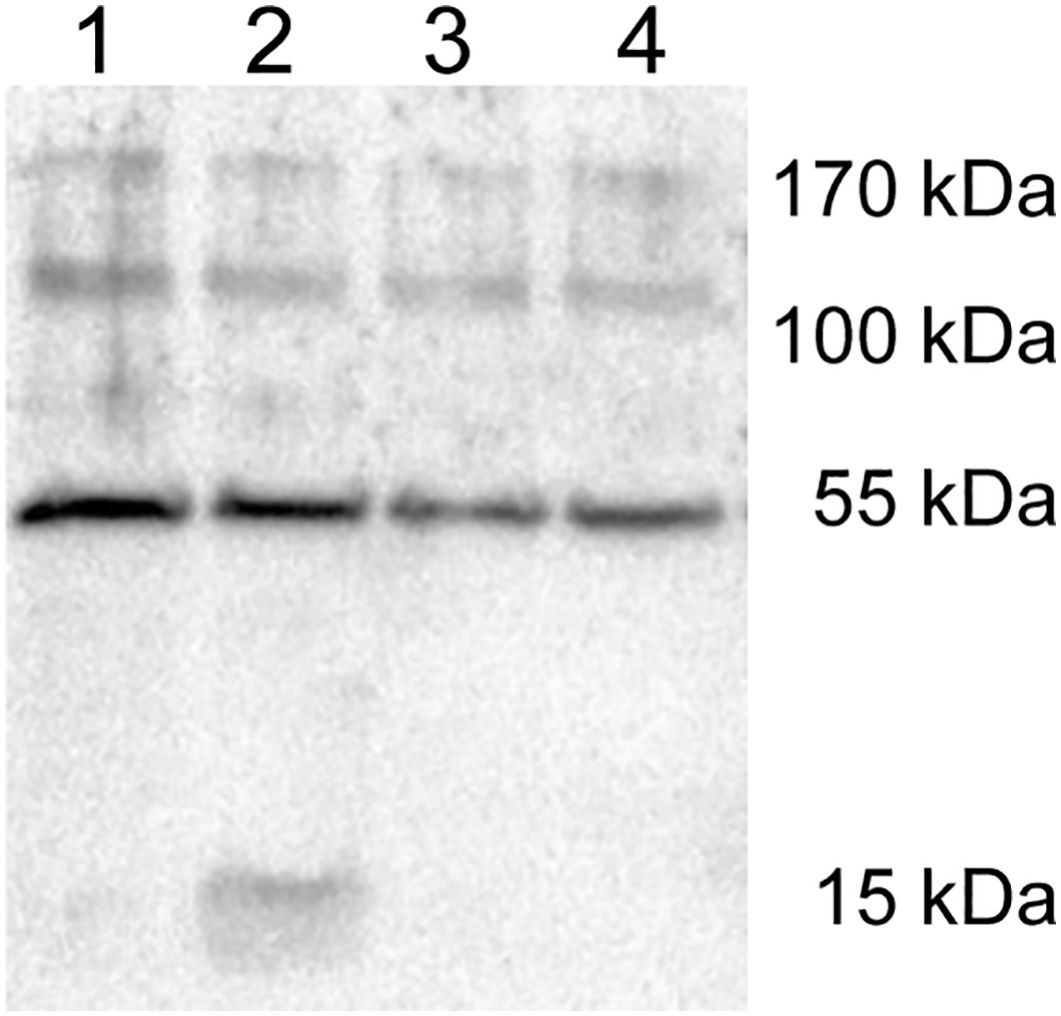
Effect of Mosd1 on Neuro-2a cells stably transfected with human APP695. Overview and detailed pictures of Neuro-2a cells, stably transfected with human APP695, are shown. Cells were treated with 0, 10 and 100 μM of Mosd1, respectively. The left panel shows untreated hAPP695-transfected Neuro-2a cells. In the middle and right panel, incubation of hAPP695-transfected Neuro-2a cells with 10 μM Mosd1 and 100 μM Mosd1 are shown. Cell viability and morphology were analyzed with a LSM 710 laser scanning microscope. Scale bars equate 50 μm.

Neuro-2a cells, stably transfected with human APP695, develop a pathologic phenotype when compared with wild type Neuro-2a cells. Wild type Neuro-2a cells accumulate and grow protrusions between each other and appear polygonal, whereas Neuro-2a cells, which are stably transfected with human APP, appear isolated, spindle shaped and show less protrusions and cell contacts (Figs 9 and 10). Applied to wild type Neuro-2a cells, Mosd1 did not alter their physiological phenotype. The cells clotted and developed protrusions and appeared polygonal (Fig 9). Added to hAPP695-transfected Neuro-2a cells, Mosd1 compensated the pathological phenotype (Fig 10). The cells developed connections and protrusions, grew denser and did partially clot, resembling the phenotype of wild type Neuro-2a cells. This effect is dose dependent, since higher concentrations of Mosd1 (10 and 100 μM) increased the development of cellular connections.

**Fig 11:**
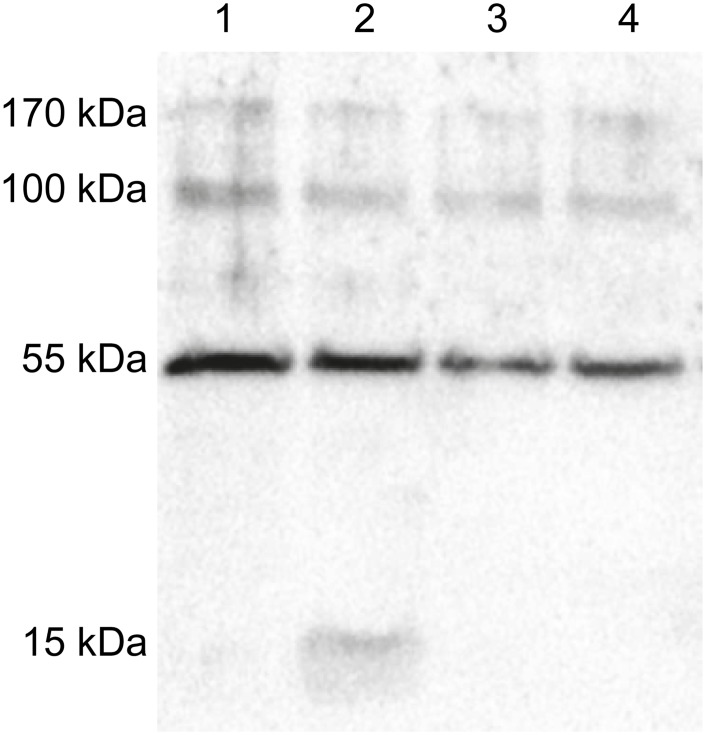
Analysis of potential γ-secretase activity alterations by Mosd1. Detection of CTFβ and β-actin by enhanced chemoluminescence on a Western blot of lysed human APP695-transfected Neuro-2a cells. Human APP695-transfected Neuro-2a cells were grown in 24-well plates for 24 hours. Cells were incubated with DMSO (lane 1), DAPT (lane 2), 10 μM Mosd1 (lane 3) or 100 μM Mosd1 (lane 4) for additional 24 hours. The cells were harvested, lysed and proteins were separated by Tris-tricine SDS-PAGE. Proteins were blotted on a PVDF membrane and detected with an anti-APP-CTF antibody as well as an anti-β-actin antibody. Binding of HRP conjugated secondary antibodies was detected by transformation of enhanced chemoluminescence (ECL) substrate by HRP.

Because Mosd1 binds Aβ_1–42_ monomers, it might also bind to the respective part of APP and influence its cleavage by γ-secretase. In order to investigate this potential side activity, we analyzed whether Mosd1 influences γ-secretase activity with APP as a substrate. Neuro-2a cells, stably transfected with the gene coding for human APP695 were incubated with 10 and 100 μM Mosd1, respectively. The cells were lysed, lysates were separated by SDS-PAGE, blotted and the APP C-terminal fragment β (APP CTFβ) was detected by enhanced chemoluminescence. Both concentrations of Mosd1 showed no signs of γ-secretase activity inhibition, i.e. the γ-secretase substrate APP CTFβ was not accumulated as it is the case for the treatment with the γ-secretase inhibitor DAPT (Fig 11) [41, 42].

## Discussion

### The competitive mirror image phage display yielded Aβ_1–42_ monomer specific ligands

Our competitive mirror image phage display led to single phage clones that preferentially bind to monomeric Aβ_1–42_. Sequencing of single phage clones revealed several consensus sequences which occurred multiply. Additionally, ELISA results showed an overall specificity of most single phage clones for monomeric Aβ_1–42_ compared to Aβ_1–42_ oligomers and fibrils and non-coated wells. Several single phage clones exhibited a very strong difference in binding affinities between monomeric and aggregated Aβ_1–42_ and also the non-coated surface. We picked the most promising clone and investigated the respective D-peptide Mosd1 for further analysis.

### Mosd1 modulates Aβ_1–42_ aggregation

Via TEM and quantitative determination of Aβ_1–42_ aggregate distribution, we were able to show that coincubation of Aβ_1–42_ with Mosd1 leads to an altered aggregation pathway of Aβ_1–42_. While incubation of Aβ_1–42_ alone for 24 hours led to oligomers and fibrillar structures, coincubation with Mosd1 led to large amorphous aggregates that were shown to be non-toxic [30, 39, 40]. By combining these results with the experiment regarding interference of Mosd1 with Aβ_1–42_ aggregate size distribution mentioned above, we suggest the following mode of action. Mosd1 stabilizes Aβ_1–42_ monomers and thereby modulates the dynamic equilibrium of Aβ_1–42_ species. Toxic Aβ_1–42_ oligomers are decomposed and the resulting Aβ_1–42_ species being precipitated and converted into amorphous non-toxic aggregates instead of fibrils. According to Ladiwala *et al*., Mosd1 can be sorted into class I molecules, which convert soluble Aβ oligomers into large, non-toxic conformers [43]. In addition, Mosd1 with 33% residues being aromatic shares the overall aromatic features of compounds, which belong to this class. Indeed, also polyphenols like resveratrol and derivatives or polyphenolic flavones like kaempferol-3-O-rhamnoside are able to cause the development of large, non-toxic, off-pathway Aβ aggregates as shown in TEM, AFM and SDS-PAGE studies by several research groups [44, 45]. Additionally, Mosd1 is able to decelerate Aβ_1–42_ aggregation. Therefore, Mosd1 is able to reduce the impact of already formed Aβ_1–42_ seeds on monomeric Aβ_1–42_ and their conversion into toxic aggregates as shown by Seeding ThT Assay.

According to cell culture experiments, aggregates generated by coincubation of Aβ_1–42_ with Mosd1 are not toxic to PC-12 cells, whereas, the mixture of Aβ_1–42_ species not treated with Mosd1 showed significant cellular toxicity. Other compounds, which consist of aromates or include aromatic side chains, were also reported to reduce Aβ induced cell toxicity [43, 45, 46]. This might be contributed to the binding to Aβ_1–42_ monomers and prevention of their aggregation as well as to the conversion of toxic oligomeric Aβ_1–42_ species into large non-toxic species as shown in the latter experiments.

Additionally, we were able to show that human APP695 transfected Neuro-2a cells, treated with Mosd1, appear healthier compared to untreated cells, which develop a pathological phenotype. Mosd1 is able to reverse the effects of human APP695 expression and its cleavage products like reduced cell contacts and a lack of cellular protrusions.

Despite the fact that Mosd1 reduces Aβ_1–42_ toxicity by modulating the equilibrium of Aβ_1–42_ species, it needs to be assured, that the compound is safe and does not interfere with related but physiological relevant pathways. For example, γ-secretases are involved in the cleavage of APP, leading subsequently to Aβ. Moreover, γ-secretases are also involved in cleavage of transmembrane Notch and therefore interfere with Notch signaling pathways. Alterations within the Notch pathway can lead to severe side effects [47, 48]. According to our results Mosd1 has no effect on γ-secretase activity.

Given the usefulness of mirror image phage display to obtain stable D-peptides for synthetically available targets, we wonder why there have been only a limited number of reports describing such approaches [49–52] and those described in reviews [26, 34, 53].

## Conclusion

Taken together, we have established a novel competitive mirror image phage display for the selection of D-enantiomeric peptides, which bind specifically to monomeric Aβ_1–42_. Using non-biotinylated SEC-derived oligomers and DGC-derived HMW aggregates and fibrils of Aβ_1–42_ as counterselective agents, we were able to enrich phages, which bind specifically to the immobilized biotinylated monomeric Aβ_1–42_.

One of our selected D-enantiomeric peptides, Mosd1, shows promising characteristics in several *in vitro* experiments. This leads to the assumption that Mosd1 is able to stabilize Aβ_1–42_ monomers, interferes with seeded growth and modulates Aβ_1–42_ aggregation towards non-toxic, amorphous aggregates and therefore rescues cells from Aβ_1–42_ derived toxicity and reverses pathological phenotypes from hAPP-transfected neuronal cells.

Mosd1 also does not affect γ-secretase function which makes it safer and more precise than γ-secretase modulating compounds. Its small size should facilitate blood-brain-barrier transfer and the D-enantiomeric conformation enables high proteolytic stability. Further investigations including affinity studies and epitope mapping should provide more information about the mechanism how Mosd1 modulates Aβ_1–42_.

We were able to show that a competitive mirror image phage display is a straightforward method to select compounds, which are Aβ_1–42_ monomer specific and able to modulate Aβ_1–42_ aggregation towards non-toxic species and therefore exhibit high therapeutic potential.
