# Correction: Competitive Mirror Image Phage Display Derived Peptide Modulates Amyloid Beta Aggregation and Toxicity

**DOI:** 10.1371/journal.pone.0159470

**Published:** 2016-07-12

**Authors:** Stephan Rudolph, Antonia Nicole Klein, Markus Tusche, Christine Schlosser, Anne Elfgen, Oleksandr Brener, Charlotte Teunissen, Lothar Gremer, Susanne Aileen Funke, Janine Kutzsche, Dieter Willbold

The images for Figs 7, 9 and 10 appear incorrectly in the published article. The figure captions are correct. Please see the correct Figs 7, 9 and 10 and their captions here.

**Fig 7:**
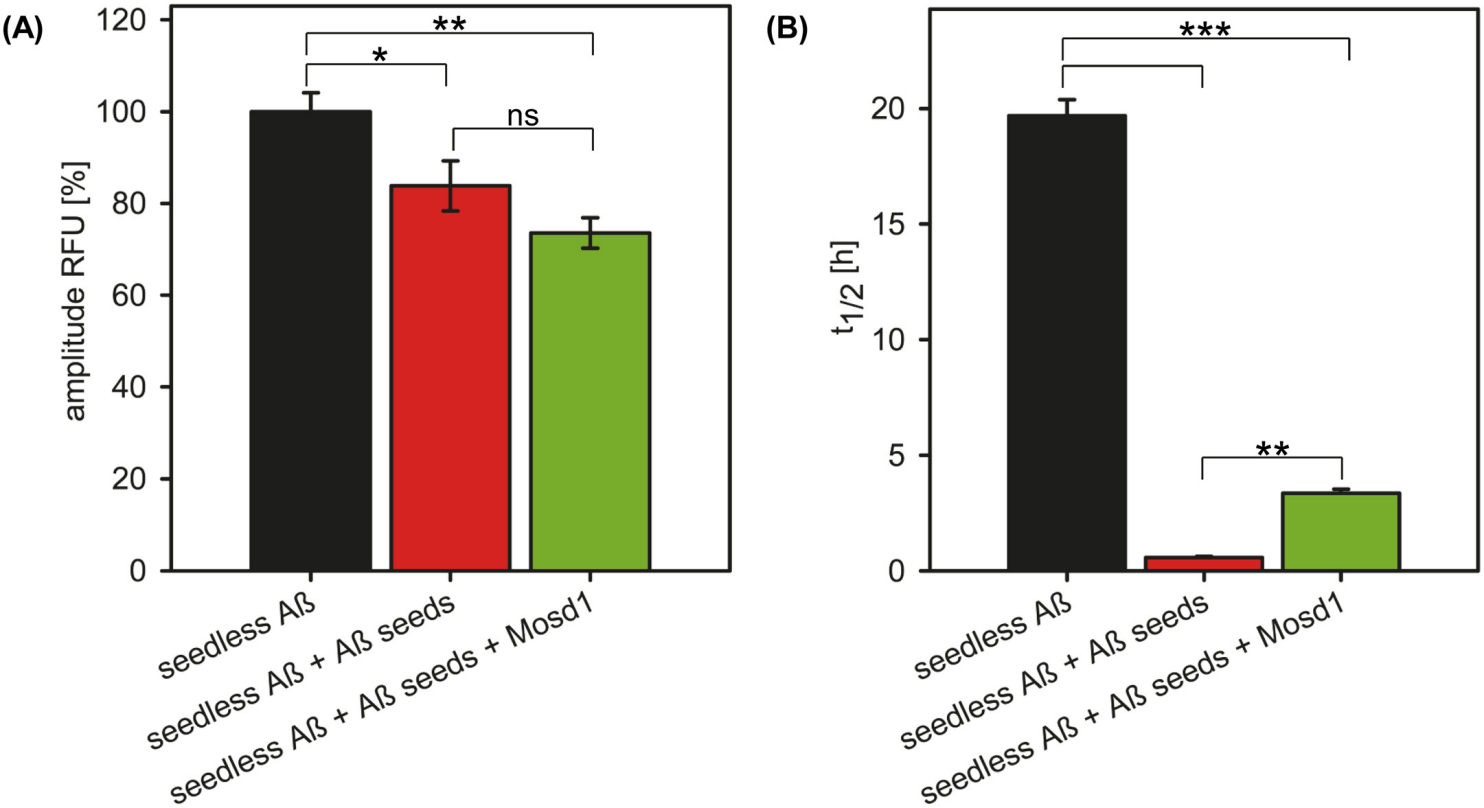
Reduction of seeded Aβ_1–42_ growth and fibrillar Aβ_1–42_ content. Seedless Aβ_1–42_ was incubated alone (black) or together with fibrillary Aβ_1–42_ seeds previously incubated with (green) or without (red) a fivefold molar excess of Mosd1. ThT (20 μM) was added to each sample in order to measure fibrillar content. The data were fitted with an asymmetric five parameter fit. The (A) amplitude of relative fluorescence (RFU) of fibrillated seedless Aβ_1–42_ served as 100% to which the other values were normalized. The (B) half-life (t_1/2_), displaying the point in time, when half of the maximum ThT signal (i.e. fibrillary content) was reached. Statistical significance was determined by one-way ANOVA. Error bars display SEM. ns: p > 0.05; *: p ≤ 0.05; **: p ≤ 0.01; ***: p ≤ 0.001.

**Fig 9:**
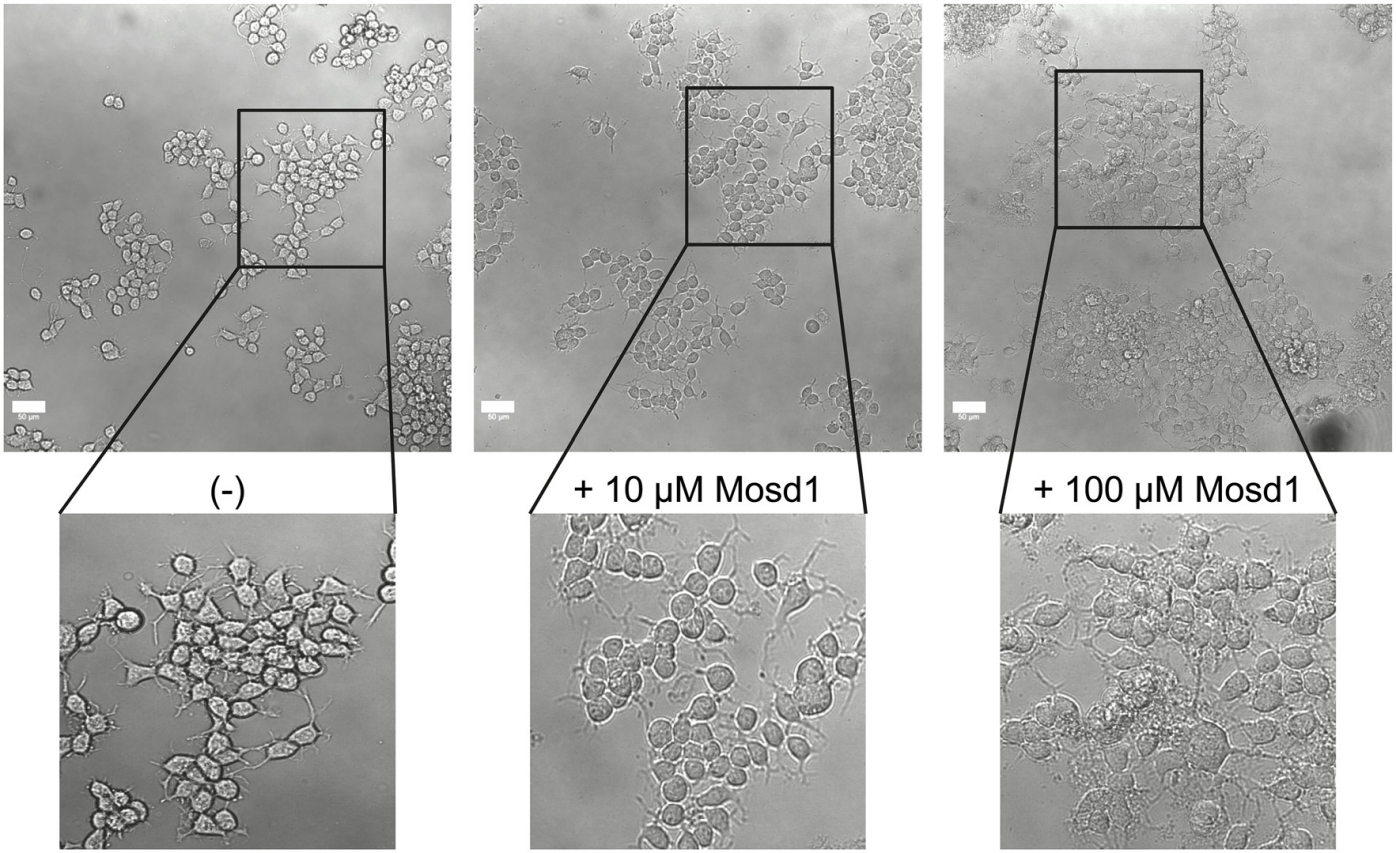
Effect of Mosd1 on Neuro-2a cells. Overview and detailed pictures of wild type Neuro-2a cells are shown. Neuro-2a cells were treated with 0, 10 and 100 μM of Mosd1, respectively. The left panel shows untreated wild type Neuro-2a cells. In the middle and right panel, incubation of wild type Neuro-2a cells with 10 μM Mosd1 and 100 μM Mosd1 are shown. Cell viability and morphology were analyzed with a LSM 710 laser scanning microscope. Scale bars equate 50 μm.

**Fig 10:**
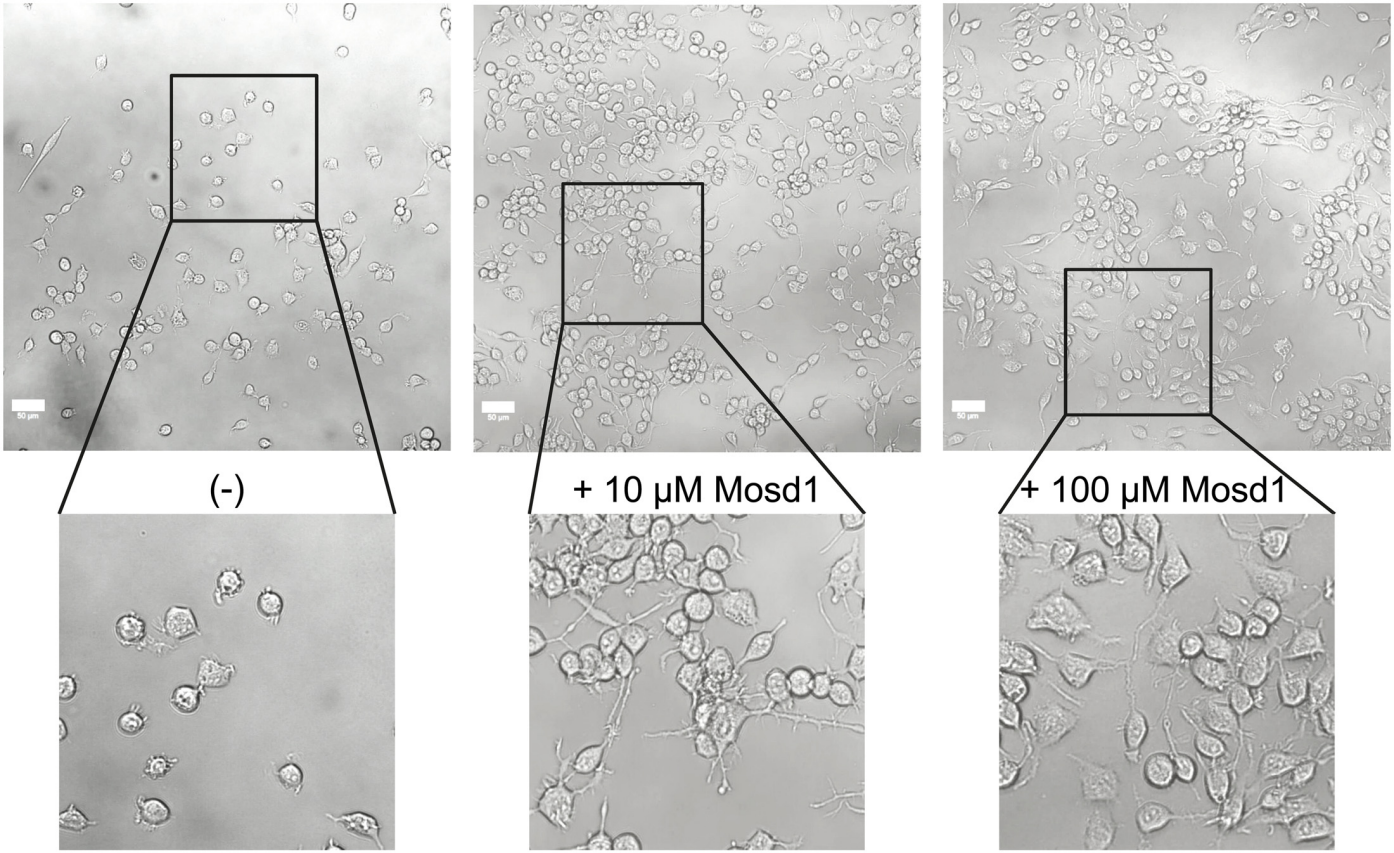
Effect of Mosd1 on Neuro-2a cells stably transfected with human APP695. Overview and detailed pictures of Neuro-2a cells, stably transfected with human APP695, are shown. Cells were treated with 0, 10 and 100 μM of Mosd1, respectively. The left panel shows untreated hAPP695-transfected Neuro-2a cells. In the middle and right panel, incubation of hAPP695-transfected Neuro-2a cells with 10 μM Mosd1 and 100 μM Mosd1 are shown. Cell viability and morphology were analyzed with a LSM 710 laser scanning microscope. Scale bars equate 50 μm.
